# Accuracy of Detecting Degrees of Lameness in Individual Dairy Cattle Within a Herd Using Single and Multiple Changes in Behavior and Gait

**DOI:** 10.3390/ani15081144

**Published:** 2025-04-16

**Authors:** Xi Kang, Junjie Liang, Qian Li, Gang Liu

**Affiliations:** 1 School of Computing and Data Engineering, NingboTech University, Ningbo 315100, China; liangjunjie0409@163.com; 2 Key Lab of Smart Agriculture Systems, Ministry of Education, China Agricultural University, Beijing 100083, China; lee55777@163.com (Q.L.); pac@cau.edu.cn (G.L.)

**Keywords:** dairy cattle, lameness detection, characteristic analysis, machine learning, computer vision

## Abstract

Lameness adversely affects the welfare and productivity of dairy cows. This study quantifies and analyzes key gait characteristics of cows with varying locomotion scores, evaluating their effectiveness for lameness detection in computer vision systems while considering individual specificity. Six key characteristics—back arch, head bob, speed, step overlap, supporting phase, and hoof step time—were analyzed to assess their distribution across different locomotion scores. Through a comparative analysis of single-parameter and multiple-parameter classification models, we quantitatively demonstrated that models using multiple characteristics significantly outperformed single-parameter models, achieving an accuracy of 84% and a Macro-F1 score of 0.81, while better accounting for individual variability. Among the characteristics, step overlap, supporting phase, and back arch showed higher relative importance in the classifiers. Back arch was a strong indicator of severe lameness, while step overlap and supporting phase were more effective for detecting mild cases. A hierarchical classification approach further improved performance by minimizing the impact of less relevant characteristics. This study highlights the importance of integrating multiple gait and posture features for robust lameness detection, providing practical insights for automated systems.

## Simple Summary

In this study, we analyzed the typical characteristics of cows with different locomotion scores, finding that using multiple characteristics for lameness detection is more effective than relying on a single trait. However, not all characteristics are suitable for individual detection, as some are more relevant at the group level. Specifically, back arch is a key indicator for severe lameness, while mild lameness is better detected through changes in leg and hoof movement characteristics like step overlap and supporting phase. A hierarchical classification approach further enhances performance by reducing individual variability.

## 1. Introduction

Lameness is a painful gait disorder in dairy cows representing significant welfare concerns for cows [1]. Lameness not only reduces milk production [2] but also impairs reproductive performance and overall cow well-being [3,4]. Early detection of lameness is crucial for timely intervention, which can prevent further complications and improve animal welfare [5]. Traditional methods of detecting lameness primarily rely on visual observations [6]. However, these approaches are time-consuming, labor-intensive, and subject to observer bias. To address these limitations, various automatic lameness detection methods have been explored, including the use of pressure sensors [7,8,9], accelerometers [10,11], and computer vision technologies [12,13,14,15,16,17], which have garnered increasing attention in recent studies. Computer vision technologies have gained attention for this application as they are relatively inexpensive, non-intrusive, and scale well with large herds.

Lameness is typically defined as a deviation in gait resulting from pain or discomfort caused by hoof or leg injuries or diseases [18]. Compared with healthy cows, lame cows exhibit distinct gait abnormalities, such as walking with an arched back and uneven movement [19]. These adjustments in posture and locomotion are believed to help alleviate pain, offering key indicators for lameness detection [18]. Previous studies have utilized computer vision systems to capture and analyze these motion characteristics, leading to the development of lameness detection algorithms [12,20]. These systems offer advantages such as ease of data collection and simple algorithm implementation [21]. However, detecting lameness in cows remains challenging due to individual specificity, such as differences in pain tolerance and locomotion characteristics among cows [22,23,24,25]. Thus, there is a growing need to develop more comprehensive lameness detection methods that consider multiple characteristics simultaneously, leveraging the integration of diverse gait and posture characteristics to enhance algorithm robustness and mitigate the impact of individual specificity.

Zhao [25] analyzed the head–hoof linkage pattern of dairy cows based on the curves of hoof movement and head swing. Li [26] proposed a spatiotemporal energy network that more comprehensively captures walking patterns by compressing walking videos into gait energy images and history energy images. However, the proposed method models the entire process of cow walking without extracting finer lameness features. Furthermore, research demonstrating the advantages of using multiple locomotion characteristics for lameness detection remains limited. Russello [15] examined six locomotion traits—back posture, head bobbing amplitude, tracking distance, stride length, stance duration, and swing duration—and found that using multiple traits improved classification accuracy compared with relying on a single characteristic. However, this study did not explore the impact of mild versus severe lameness or provide insights into why multiple characteristics lead to better detection.

In summary, numerous studies have acknowledged that individual variability poses a challenge to the precise detection of lameness in dairy cows. However, there is a scarcity of research that quantifies the impact of individual variability on the characteristics associated with bovine lameness, and empirical data demonstrating how this issue affects detection outcomes is limited. Some studies have suggested the use of multiple characteristics to detect lameness in an effort to mitigate the influence of individual variability. Nonetheless, there is a paucity of research investigating whether an increase in the number of characteristics correlates with improved detection efficacy, or whether an optimal combination of characteristics could yield superior results. Furthermore, the question of whether different characteristics are more effective for classifying varying degrees of lameness remains largely unexplored.

In light of these challenges, our article pursues two primary objectives. The first is to provide a clear and quantitative assessment of the variability in lameness characteristics under the influence of individual specificity and to examine whether the use of multiple characteristics for lameness detection is more effective than reliance on a single characteristic in such contexts. The second objective is to conduct an in-depth analysis of these characteristics to explore whether an increased number of characteristics leads to better detection outcomes, whether an optimal combination of characteristics can enhance performance, and how different characteristics affect the classification of varying degrees of lameness. This research will provide valuable theoretical insights to guide the development of more accurate and robust lameness detection methods for dairy cows.

## 2. Materials and Methods

### 2.1. Image Acquisition

**Figure 1:**
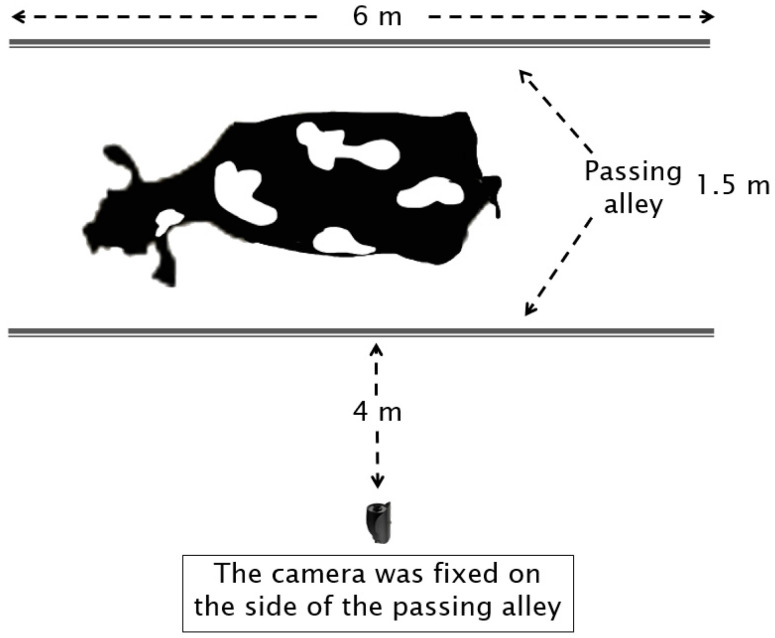
Top view of the video data collection system for walking cows.

The dataset was collected at Dingyuan Farm, located in Hebei Province, China, in September 2020. The herd consisted of 1000 lactating Holstein cows, from which 300 multiparous lactating Holstein cows were randomly selected. After excluding cows that stopped, crowded, or overlapped during the recording process, a total of 175 cows were included in the study. The experimental setup consisted of a 6 × 1.5 m passing alley situated at the exit of a milking parlor. A digital camera (Panasonic DC-GH5S, Tokyo, Japan) was mounted on a tripod positioned 4 m from the side of the passing alley (Figure 1).

The videos were recorded over three days as the cows passed individually and freely through the alley. In total, 175 video recordings were obtained, each with a duration ranging from 5 to 10 s. The video resolution was 1920 × 1080 pixels, and the camera frame rate was set to 50 frames per second. Each video captured the entire body of the cow, along with the movement periods of the cows.

Ethical approval was not required for this study, as the data collection process was entirely non-invasive. Video recordings were obtained using a stationary camera placed at a distance from the cows, ensuring that no physical contact or interference with the animals occurred.

### 2.2. Locomotion Scoring by Visual Observations

The locomotion of the cows was scored by two observers trained in locomotion scoring at the passing alley. We used a simplified locomotion scoring system to categorize the cows’ movement into three scores: Score 1 indicates cows that are not lame, Score 2 indicates cows with mild lameness, and Score 3 indicates cows with severe lameness. The description for each score is shown in Table 1. Each observer scored the cows at the passing alley located at the exit from the milking parlor and also scored the cows twice from the video recordings. If discrepancies in scoring arose, the two observers would review the video repeatedly to reach a consensus result.

### 2.3. Characteristic Parameter Acquisition

**Figure 2:**
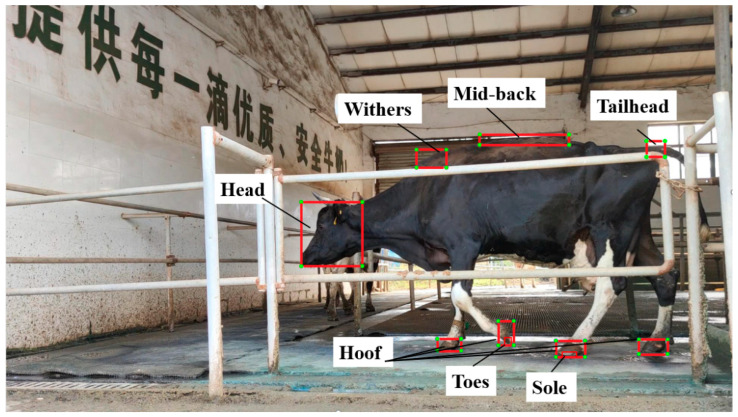
Precise location of cows’ anatomy producing data for computer analyses.

Based on established methods for detecting lameness in dairy cows, six key kinematic parameters were selected for this study: back arch, head bob, hoof step time, step overlap, speed, and supporting phase. These parameters were chosen because they comprehensively capture the head and back postures during cow locomotion, overall velocity, as well as temporal and spatial information of hoof movement phases. The computational procedures and data sources for deriving these parameters are summarized in Table 2, providing a detailed reference for their calculation. To ensure data accuracy, key body parts of the cows, essential for parameter calculation, were manually annotated. This involved marking the central coordinates of the cow’s head, withers, hooves, mid-back, and tailhead to establish reference points for parameter expression. As shown in Figure 2, every frame of the video that captured a walking cow was marked. The annotations encompassed both the location of the key body parts and specific details required for the characteristic parameter calculations. For example, we annotated the positions of the front, back, left, and right legs; the phases of the gait cycle; and the transition points of hoof contact and lift-off. These annotations enabled the calculation of temporal data such as stride duration and phase timing based on the frame rate and the sequence of marked frames. Frames with obscured body parts were interpolated using data from adjacent frames to ensure continuity and consistency in the dataset.

### 2.4. Locomotion Score Prediction

In this study, we employed machine learning classification algorithms to evaluate the predictive performance of each kinematic characteristic in classifying locomotion scores. Lameness classification was performed using three widely used machine learning algorithms: support vector machine (SVM), decision tree, and logistic regression [31,32,33]. These classifiers learn from given examples and can be trained to score lameness [15]. Multiple classifiers were employed to ensure robustness and generalizability of the results, as relying on a single classifier might lead to model-specific biases and reduce the persuasiveness of the findings. The dataset comprised 80, 66, and 29 cows with locomotion scores of 1, 2, and 3, respectively, resulting in an imbalanced class distribution. To mitigate overfitting, the data were normalized, and regularization techniques combined with fivefold cross-validation were applied throughout the detection process. Stratified sampling was employed to maintain an equal ratio of the three locomotion scores across the fivefold cross-validation subsets. These measures ensured balanced representation of each group and minimized bias due to imbalanced sample sizes. All analyses were conducted using PyCharm 3.8, with the pandas library for data manipulation, sklearn for machine learning, numpy for numerical computations, and matplotlib for visualization. The kernel of the SVM was the linear, the criterion of decision tree learning was entropy, and the logistic regression classifier used multinomial regression.

For classifiers utilizing a single characteristic parameter, we trained six instances of each algorithm type, with each instance corresponding to one of the six characteristic parameters. For each classifier, the input *x* consisted of a single characteristic parameter vector, while the output *y* represented the locomotion score of the cow. To compare the performance of locomotion score classification using multiple characteristic parameters versus a single parameter, we trained one instance of each algorithm type using all six characteristic parameters as input. Classification effectiveness at each lameness level was evaluated using sensitivity, specificity, precision, recall, and Macro-F1 scores, which were calculated using Equations (1)–(6), respectively:

(1) $$Accuracy=\frac{TP+TN}{TP+TN+FP+FN}\times 100\%$$

(2) $$Specificity=\frac{TN}{TN+FP}$$

(3) $$Sensitivity=Recall=\frac{TP}{TP+FN}$$

(4) $$\mathrm{Precision}=\frac{\mathrm{TP}}{\mathrm{TP}+\mathrm{FP}}$$

(5) $$F_{1}-{\mathrm{score}}_{i}=2\times \frac{{\mathrm{Precision}}_{i}\times {\mathrm{Recall}}_{i}}{{\mathrm{Precision}}_{i}+{\mathrm{Recall}}_{i}}$$

(6) $$\mathrm{Macro}\text{-}F_{1}=\frac{F_{1}-{\mathrm{score}}_{1}+F_{1}-{\mathrm{score}}_{2}+F_{1}-{\mathrm{score}}_{3}}{3}$$

To evaluate the overall efficacy of the classifiers, we selected accuracy and Macro-F1 as the primary performance metrics. The Macro-F_1_ score was computed by calculating the F_1_ score for each class and then taking the unweighted mean across all classes. The Macro-average is especially useful with imbalanced datasets, as all classes contribute equally to the metric.

In the cow lameness detection system, an excessive number of input parameter types is not recommended, as it may increase system complexity and elevate the likelihood of errors during parameter acquisition. Therefore, selecting a balanced set of parameters that effectively capture lameness-related features while maintaining system efficiency is crucial. To determine whether reducing the number of classification characteristics could maintain comparable performance, we evaluated the relative importance of each parameter to the classifiers. Based on these findings, we further investigated the contribution of each characteristic parameter to individual lameness detection performance.

To quantify the relative importance of the six lameness detection characteristics, we employed a weight-based feature importance analysis method [34,35]. First, all characteristic data were standardized (mean = 0, variance = 1) to ensure comparability across features with different units and scales. The weight coefficients or characteristic importance scores of classifiers were then extracted. To ensure the stability and generalizability of the results, characteristic importance values were computed using fivefold stratified cross-validation and averaged as the final importance scores.

## 3. Results

### 3.1. Descriptive Analysis and Correlation

**Figure 3:**
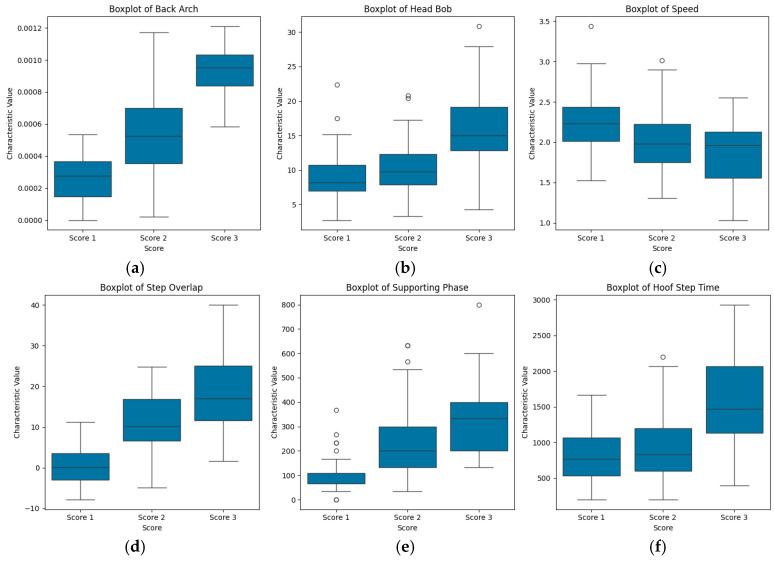
Box plots of the 6 parameters: back arch (**a**), head bob (**b**), speed (**c**), overlap (**d**), supporting phase (**e**), and hoof step time (**f**) versus the locomotion scores of 175 cows.

Figure 3 presents the box plots of the six parameters across the three locomotion scores. Specifically, Figure 3a–f illustrate the distributions of back arch, head bob, speed, step overlap, supporting phase, and hoof step time for cows with locomotion scores of 1, 2, and 3. The analysis focused on identifying demarcation thresholds in characteristic distributions across locomotion scores to assess classification utility. In Figure 3a,b, the distributions of back arch and head bob exhibit overlap between Scores 1 and 2, but both parameters show statistically significant separation from Score 3. In Figure 3d,e, the distributions of step overlap and supporting phase exhibit overlap between Scores 2 and 3, but both parameters show statistically significant separation from Score 1. Figure 3c,f reveal that the distributions of speed and hoof step time overlap across all three locomotion scores.

**Figure 4:**
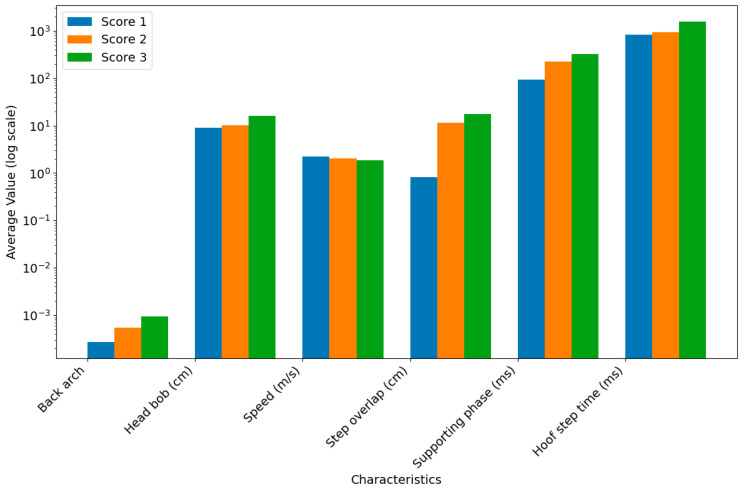
The average values of six characteristic parameters of cows with different locomotion scores.

The average values of the six characteristic parameters for cows across different locomotion scores are presented in Table 3 and Figure 4. The values of the lameness-related characteristic parameters exhibited consistent trends across different locomotion scores. This suggests that these characteristics are closely associated with the movement patterns of lame cows, further validating findings from previous studies. However, the applicability of these characteristics for individual cow lameness recognition requires further analysis on a case-by-case basis. These findings highlight the potential of these characteristics as indicators of lameness severity at a population level. However, individual variability in movement patterns may limit their direct application to lameness recognition in individual cows, necessitating additional research to refine their utility in practical settings.

### 3.2. Classification Accuracy

The cross-validated classification accuracies on the test dataset, obtained using both single-parameter and multiple-parameter approaches, are summarized in Table 4. In the table, we focused on comparing the accuracy and Macro-F1 scores of classifiers with different input configurations of single parameters and multiple parameters. The classification accuracy varied significantly across the six parameters and among the different algorithms. Compared with the single-parameter classification results, the multiple-parameter approach demonstrated superior performance and stability, achieving an accuracy of approximately 84% and a Macro-F1 score of 0.81. These results suggest that combining multiple parameters enhances both the accuracy and robustness of lameness classification, making it a more reliable approach for practical applications compared with single-parameter methods.

The relative importance of the six characteristic parameters is presented in Table 5. The relative importance of the characteristic parameters varied across different classifiers. Overall, the top three most important characteristic parameters were consistently step overlap, supporting phase, and back arch, whereas the remaining three parameters exhibited relatively lower importance. This consistency in the top three parameters highlights their potential as robust indicators for lameness detection, while the lower importance of the remaining parameters suggests their limited utility in classification tasks.

**Figure 5:**
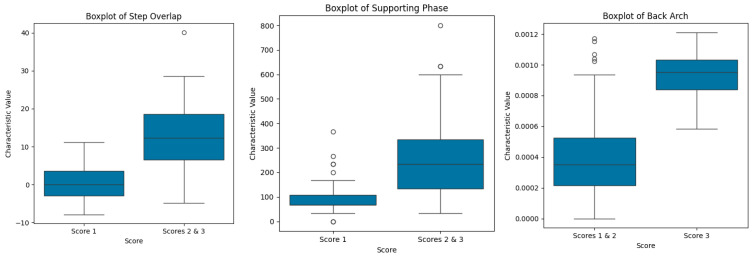
The distributions of the step overlap, supporting phase, and back arch characteristics.

We visualized the distributions of the step overlap and supporting phase characteristics for healthy and lame cows, along with the distribution of the back arch characteristic for healthy and mildly lame cows compared with severely lame cows, as shown in Figure 5. Among them, there were significant differences in the distributions of the step overlap and supporting phase characteristics between cows with Score 1 and lame cows with Scores 2 and 3, particularly in the step overlap. The median for the combined Scores 2 and 3 group was approximately 10, significantly higher than that of the Score 1 group, which was around −2.5. The interquartile range (IQR) for the Scores 2 and 3 group spans from about 5 to 15, whereas the IQR for the Score 1 group ranges from approximately −5 to 0, indicating a clear numerical disparity in the step overlap characteristic values.

Additionally, the back arch characteristic distribution showed a significant difference between cows with Score 3 and those with Scores 1 and 2. The median for the Score 3 group was approximately 0.0010, significantly higher than that of the Scores 1 and 2 group, which was around 0.0005. The IQR for the Score 3 group ranged from about 0.0009 to 0.0011, while the IQR for the Scores 1 and 2 group spanned from approximately 0.0004 to 0.0006.

## 4. Discussion

### 4.1. Individual Specificity

Many studies on lameness detection have mentioned cows’ individual specificity and considered it a challenge hindering accurate detection, as variations in gait patterns and physiological responses among individual cows can significantly affect the performance of detection algorithms [22,23,24,25]. However, few studies have quantified the extent of individual specificity, making it challenging to comprehensively assess its impact on lameness detection. This issue is less pronounced during manual observation, as observers can subjectively identify uneven gait patterns without requiring precise measurements of individual leg swing phases. In contrast, computer vision-based detection techniques exacerbate this issue, as they depend on quantitative data derived from cows’ walking patterns to compute lameness-related characteristics. Cow individual specificity introduces significant variations in data among different cows, ultimately compromising the accuracy of lameness detection results. These findings underscore the need for developing adaptive algorithms that account for individual variability, thereby improving the robustness and reliability of computer vision-based lameness detection systems.

We propose that the key to addressing this issue lies in the development of specific quantitative metrics, akin to the locomotion scores used in manual observation, to objectively describe healthy cows and enable automated lameness assessment. For instance, while current studies indicate that healthy cows typically do not arch their backs during walking [36,37], there is no well-defined threshold to distinguish a ‘level-back posture’ from an ‘arched-back posture’, nor a clear criterion to determine when an arched-back posture becomes evident. To address this challenge, the first step is to understand cow individual specificity. It is essential to identify characteristics that exhibit significant individual variability and those that overlap across cows with different lameness severities. Ultimately, the goal is to identify characteristics with minimal individual variability that can reliably detect lameness, thereby enhancing the effectiveness of computer vision-based lameness detection technologies.

The mean values of each characteristic, as shown in Table 3 and Figure 4, vary significantly across locomotion scores, demonstrating that cows with different locomotion scores exhibit measurable differences in these characteristics at the population level. However, as shown in Figure 3, none of the characteristics are completely separable across the three locomotion scores, indicating that individual cows with different locomotion scores may not consistently exhibit these characteristic differences due to individual variability. This underscores the limitation of relying on a single characteristic for lameness classification, as individual variability can lead to substantial misclassification errors.

### 4.2. Multiple-Parameter Detection

Single-characteristic detection of lameness is significantly influenced by individual variability, especially when detecting mild lameness, where subtle gait changes may vary widely among cows. Therefore, many studies have investigated the use of multiple characteristics for lameness detection to mitigate the effects of individual variability, thereby enhancing the comprehensiveness and robustness of the detection system [38,39]. However, few studies have provided empirical evidence to validate the effectiveness of this approach, particularly in the context of mild lameness detection. As shown in Table 4, the results indicate that multiple-parameter detection significantly enhances detection accuracy and reliability.

Under conditions of hoof pain and discomfort, different cows exhibited distinct abnormal gait characteristics compared with normal cows, with cows displaying mild lameness showing a wider range of variability in these characteristics. In this context, multiple-parameter detection is more effective in accurately identifying lame cows, especially those with mild lameness, due to its ability to capture a broader spectrum of gait abnormalities. The results further clarify that, in our comparative experiments, the multiple-parameter detection method achieved a significant improvement in sensitivity, particularly for detecting cows with mild lameness, highlighting its practical utility.

Although the multiple-parameter detection method outperformed the single-parameter approach in this study, it does not necessarily imply that adding more parameters will lead to further performance improvements. While the multiple-parameter approach demonstrated superior performance in this study, the inclusion of additional parameters may introduce redundancy or noise, potentially diminishing the detection accuracy. Therefore, careful selection and optimization of parameters are essential to maximize the effectiveness of lameness detection systems.

As shown in Table 5, the top three most significant characteristic parameters, identified through feature importance analysis, were step overlap, supporting phase, and back arch. Using these three characteristics as inputs, we developed an SVM classification model with a linear kernel, achieving an accuracy of 87% and a Macro-F1 score of 0.84. This performance surpassed that of the classifier trained on all six characteristics. This finding demonstrates that while multiple characteristics can reduce the impact of individual variability, not all characteristics are equally effective for lameness detection.

### 4.3. Characteristics and Detection Methods

As shown in Figure 3, certain characteristics demonstrate greater separation in their distributions across different locomotion scores. For instance, the back arch characteristic shows a clear separation in the distribution of Score 3 compared with the other two scores (Score 1 and Score 2), while the step overlap and supporting phase characteristics exhibit significant separation in the distribution of Score 1 compared with the other two scores. Figure 5 visualizes the distributions of the step overlap and supporting phase characteristics for healthy and lame cows. The distributions of each characteristic show significant separation between the two groups, emphasizing their ability to distinguish between different degrees of lameness. These observations suggest that these characteristics hold promise for distinguishing between different locomotion scores, particularly in the early detection of lameness. This further validates that the early lameness in dairy cows is primarily characterized by leg and hoof changes; it is important to note that subtle changes in leg kinematics may also provide valuable insights for early lameness detection and the potential of gait variables for application in early lameness detection systems [40,41,42]. However, significant distributional differences existed in the back arch characteristic between healthy/mildly lame cows and severely lame cows. The back arch characteristic, while occasionally observable in mild lameness cases, demonstrates inconsistent occurrence and minimal manifestation. In contrast, severe lameness consistently exhibits markedly prominent back arching. This explains why, in the classification results, the back arch characteristic demonstrated the highest sensitivity specifically for Score 3 cases compared with other characteristics.

Based on these findings, we developed a combined SVM classifier using a hierarchical approach. First, we used the back arch characteristic to classify severely lame cows, followed by the step overlap and supporting phase characteristics to distinguish between healthy and mildly lame cows among the non-severely lame cases. We found that this hierarchical approach achieved superior performance compared with the previously mentioned method of directly performing three-class classification using all three characteristics, particularly in terms of accuracy and Macro-F1 scores. The first classifier achieved an accuracy of 91% and a Macro-F1 score of 0.95, and the second classifier achieved an accuracy of 93% and a Macro-F1 score of 0.93, yielding an overall accuracy of 90%. These results suggest that a multi-step, hierarchical classification approach leveraging different characteristics based on their discriminative properties is an effective strategy. This approach minimizes the impact of individual variability by isolating the influence of less relevant characteristics.

Our findings are consistent with those of Russello et al. (2024), demonstrating the effectiveness of multiple locomotion traits for lameness detection [15]. Integrating multiple locomotion traits enhances the robustness of lameness detection systems, particularly by reducing misclassification rates caused by individual variability among cows. However, notable differences exist in the treatment of head bobbing and the classification methodologies employed. While Russello et al. (2024) [15] classified cows into binary categories (lame vs. non-lame), our study differentiated between mild and severe lameness. This distinction may explain the lower sensitivity of head bobbing in our results, as it is less pronounced in mild cases. Specifically, head bobbing was more pronounced in severe lameness but exhibited greater variability in mild cases, underscoring the importance of considering lameness severity when interpreting this trait.

Additionally, it is important to note that our study is based on data from 175 cows, which may limit the generalizability of the results. The relatively small sample size could restrict the broader applicability of our findings. To address this limitation, future studies should aim to expand the sample size by including a more diverse and larger population of cows. This would not only improve the robustness of the results but also offer more comprehensive insights into the mechanisms and early detection strategies for lameness.

## 5. Conclusions

In this study, we demonstrated that combining multiple gait characteristics significantly improves classification accuracy and robustness compared with relying on a single characteristic. However, incorporating too many characteristics does not necessarily lead to improved classification performance. We recommend including key characteristics such as back arch for posture analysis and spatiotemporal leg characteristics like step overlap and supporting phase for gait analysis. Additionally, a multi-step, hierarchical classification approach, leveraging different characteristics based on their discriminative properties, can effectively reduce the impact of individual variability and improve detection accuracy. In future work, we plan to explore advanced feature selection techniques and further optimize the multi-step classification framework to enhance lameness detection performance.

## Figures and Tables

**Table 1:** Description of a 3-point lameness score for dairy cows.

Score	Description
1	The cow walks with a level-back posture. The gait is normal. No signs of head bob when the cow is walking.
2	In most cases, the back is arched when the cow is walking. The gait might be slightly uneven and the cow may walk with short strides. In most cases, there are no signs of head bob when walking.
3	The back is visibly arched when the cow is walking. The cow is obviously lame on 1 or more legs. The cow is unable, unwilling, or very reluctant to bear weight on the affected leg. In most cases, head bob will be evident when walking.

**Table 2:** The computational procedures and data sources.

Reference	Summary of the Methods	Outcome	Expression	Interpretation
Poursaberi [22]	Three points (hip point, shoulder point, middle point) were used to measure the curvature of the back, and two thresholds were used to implement the classification.	96% correct rate of classification	$k=\frac{1}{R}$	where R denotes the radius of the circle passing through three points, and k denotes the curvature of the back.
Flower [27]	Continuous 100-unit scales were used to assessed 6 gait attributes: back arch, head bob, tracking-up, joint flexion, asymmetric gait, and reluctance to bear weight.	92% correct rate of classification	-	-
Russello [15]	The amplitude of the vertical movement of the forehead keypoint was used as a measure of head bobbing.	Head bobbing amplitude displayed a clear demarcation between the normal and lame classes.	$H=max(h_{hi}-h_{li})\;(i=1,2,\cdots ,$ n)	where H denotes the maximum of the cow’s head amplitude, *h_hi_* denotes the highest location of the center of the cow’s head, *h_li_* denotes the lowest location of the center of the cow’s head, and n denotes the number of times the cow’s head swings.
Zillner [28]	Speed of cows was taken by a standard stopwatch, and statistical data analysis was carried out using SPSS version 23.	The sensitivity was 71.43%, and the specificity was 78.57%.	$v=\frac{s}{t}$	where v denotes the walking speed, s is the length of the test track, and t denotes the time needed to cover the test track.
Song [12]	Linear relation between step overlaps and human visual locomotion scores was analyzed.	The step overlaps had a positive linear relationship to the visual locomotion scores.	${∆}_{Left}=X_{\;FL}-X_{\;HL}$ ${∆}_{Right}=X_{\;FR}-X_{\;HR}$ $∆=max({∆}_{Left},{∆}_{Right})$	${∆}_{Left}$ denotes the step overlap of the left side, and ${∆}_{Right}$ denotes the step overlap of the right side. ∆ denotes the maximum values of ${∆}_{Left}\;\mathrm{and}\;{∆}_{Right}$.
Kang [29]	The Spearman rank correlation coefficient was calculated for the locomotion score and the difference in the supporting phases.	The correlation coefficient was 0.864.	$\mathrm{Supporting}\;\mathrm{phase}=T_{Lift}-T_{Land}$	where $T_{Lift}$ is the time of hoof toe being com-pletely lifted off the ground, and $T_{Land}$ is the time of hoof sole being fully loaded.
Bahr [30]	The correlation between hoof step time and visual locomotion scores was analyzed.	The correlation coefficient was 0.84.	${∆}_{Step}=T_{touch}-T_{lift}$ ${∆}_{hoofsteptime}=T_{fs}+T_{ss\;}$	where ${∆}_{Step}$ denotes the step time of the hoof, $T_{touch}$ denotes the time that the hoof touches the ground, $T_{lift}$ denotes the time that the hoof is lifted off the ground, $T_{fs}$ denotes the time of the first stride, $T_{ss}$ denotes the time of the second stride, and ${∆}_{hoof\;step\;time}$ denotes the corresponding times of both strides.

**Table 3:** The average values of six characteristic parameters of cows with different locomotion scores.

Characteristic	Back Arch	Head Bob (cm)	Speed (m/s)	Step Overlap (cm)	Supporting Phase (ms)	Hoof Step Time (ms)
Average values of Score 1	2.7 × 10^−4^	9.0	2.2	0.8	93.1	821.6
Average values of Score 2	5.4 × 10^−4^	10.1	2.0	11.3	225.4	941.8
Average values of Score 3	9.5 × 10^−4^	15.8	1.9	17.3	318.0	1576.9

**Table 4:** Classification results of the six single parameters and multiple parameters by classifiers.

Characteristic	Algorithm	Accuracy (%)	Sensitivity of Score 1	Specificity of Score 1	Sensitivity of Score 2	Specificity of Score 2	Sensitivity of Score 3	Specificity of Score 3	Macro-F_1_
Back arch	Support vector machine	74	0.84	0.74	0.48	0.79	**0.63**	0.96	0.69
	Decision tree learning	66	0.69	0.78	0.48	0.71	**0.77**	0.91	0.65
	Logistic regression	75	0.84	0.67	0.48	0.79	**0.63**	0.96	0.69
Head bob	Support vector machine	71	0.38	0.66	**0.01**	0.99	0.38	0.98	0.37
	Decision tree learning	52	0.61	0.62	**0.47**	0.72	0.53	0.88	0.53
	Logistic regression	80	0.61	0.61	**0.01**	0.99	0.35	0.98	0.45
Speed	Support vector machine	71	0.65	0.64	0.23	0.85	0.01	0.99	0.41
	Decision tree learning	44	0.55	0.67	0.45	0.58	0.28	0.89	0.43
	Logistic regression	71	0.55	0.75	0.02	0.98	0.01	0.99	0.30
Trackway overlap	Support vector machine	79	0.81	0.81	0.62	0.73	0.10	0.90	0.62
	Decision tree learning	53	0.65	0.82	0.56	0.68	0.37	0.82	0.53
	Logistic regression	69	0.81	0.80	0.55	0.75	0.20	0.97	0.59
Supporting phase	Support vector machine	77	0.83	0.76	0.58	0.73	0.01	0.99	0.58
	Decision tree learning	62	0.75	0.81	0.46	0.73	0.18	0.92	0.53
	Logistic regression	50	0.86	0.74	0.50	0.76	0.10	0.97	0.51
Hoof step time	Support vector machine	80	0.26	0.80	0.05	0.93	0.30	0.98	0.31
	Decision tree learning	54	0.69	0.54	0.36	0.65	0.40	0.93	0.50
	Logistic regression	79	0.63	0.56	0.01	0.99	0.35	0.99	0.45
Multiple parameters	Support vector machine	**85**	0.95	0.93	0.74	0.89	0.63	0.95	**0.81**
	Decision tree learning	**84**	0.88	0.93	0.82	0.84	0.73	0.97	**0.82**
	Logistic regression	**82**	0.94	0.93	0.69	0.88	0.72	0.94	**0.80**

**Table 5:** The relative importance of six characteristic parameters of the classifiers.

Algorithm	Back Arch	Head Bob	Speed	Step Overlap	Supporting Phase	Hoof Step Time
Support vector machine	0.89	0.07	0.34	**1.86**	1.17	0.19
Decision tree learning	0.320	0.102	0.084	**0.326**	0.111	0.057
Logistic regression	1.17	0.16	0.42	**2.30**	1.60	0.16

## Data Availability

No new data were created.
